# Abnormal Spontaneous Brain Activity in Women with Premenstrual Syndrome Revealed by Regional Homogeneity

**DOI:** 10.3389/fnhum.2017.00062

**Published:** 2017-02-13

**Authors:** Hai Liao, Yong Pang, Peng Liu, Huimei Liu, Gaoxiong Duan, Yanfei Liu, Lijun Tang, Jien Tao, Danhong Wen, Shasha Li, Lingyan Liang, Demao Deng

**Affiliations:** ^1^Department of Radiology, First Affiliated Hospital, Guangxi University of Chinese MedicineNanning, China; ^2^Department of Acupuncture, First Affiliated Hospital, Guangxi University of Chinese MedicineNanning, China; ^3^Life Science Research Center, School of Life Science and Technology, Xidian UniversityXi’an, China; ^4^Department of Teaching, First Affiliated Hospital, Guangxi University of Chinese MedicineNanning, China

**Keywords:** premenstrual syndrome, resting state, fMRI, regional homogeneity, brain

## Abstract

**Background**: Previous studies have revealed that the etiologies of premenstrual syndrome (PMS) refer to menstrual cycle related brain changes. However, its intrinsic neural mechanism is still unclear. The aim of the present study was to assess abnormal spontaneous brain activity and to explicate the intricate neural mechanism of PMS using resting state functional magnetic resonance imaging (RS-fMRI).

**Materials and Methods**: The data of 20 PMS patients (PMS group) and 21 healthy controls (HC group) were analyzed by regional homogeneity (ReHo) method during the late luteal phase of menstrual cycle. In addition, all the participants were asked to complete a daily record of severity of problems (DRSP) questionnaire.

**Results**: Compared with HC group, the results showed that PMS group had increased ReHo mainly in the bilateral precuneus, left inferior temporal cortex (ITC), right inferior frontal cortex (IFC) and left middle frontal cortex (MFC) and decreased ReHo in the right anterior cingulate cortex (ACC) at the luteal phase. Moreover, the PMS group had higher DRSP scores, and the DRSP scores positively correlated with ReHo in left MFC and negatively correlated with ReHo in the right ACC.

**Conclusion**: Our results suggest that abnormal spontaneous brain activity is found in PMS patients and the severity of symptom is specifically related to the left MFC and right ACC. The present findings may be beneficial to explicate the intricate neural mechanism of PMS.

## Introduction

Premenstrual syndrome (PMS) refers to a series of cycling and relapsing physical, emotional, cognitive and behavioral symptoms that regularly recur during the late luteal phase of each menstrual cycle and relieve soon after the onset of menses (Tacani et al., 2015). It is estimated that more than 80% of women are affected by PMS (Halbreich, 2003). The symptoms of PMS are often mild, but many patients present gradually worsening symptoms within 10 years (Freeman, 2003), and approximately 3%–10% of PMS women suffer from severe syndrome associated with substantial distress or functional impairment which eventually reach the criteria for premenstrual dysphoric disorder (PMDD; Hamaideh et al., 2014; Ryu and Kim, 2015). PMS has a significant negative effect on woman’s quality of life, and disturbs family relationships, work, productivity, social activity and sexual relationships (Freeman, 2003; Halbreich et al., 2003). It is also an important risk factor for postpartum depression (Buttner et al., 2013). However, no characteristic symptoms and signs occur, nor is a recognizable physiological and anatomical factor identified in PMS. Thus, it is necessary to pay more attention to understanding the underlying mechanism of PMS.

Regarding the etiology mechanism of PMS, there exist a great argument on gonadal hormones (estrogen and progesterone), gene, psychosocial factors and certain central nervous system (CNS) pathways (Duvan et al., 2011; Rapkin and Akopians, 2012; Barth et al., 2015; Hantsoo and Epperson, 2015). The evidence from previous brain imaging studies has showed that PMS exists CNS dysfunctions. In the animal model, PMS is associated with dysregulation of hippocampus (Barth et al., 2014; Gao et al., 2014). As to the patients, the abnormalities of brain functional activity are also involved in PMS. Based on the single photon emission computed tomography (SPECT), the decreases of regional cerebral blood flow (rCBF) were reported to be located in the temporal lobes at luteal phase compared with the follicular phase in PMS patients (Buchpiguel et al., 2000). Liu Q. et al. (2015) found that compared with healthy subjects, women with PMS during luteal phase displayed decreased connectivity in the middle frontal gyrus and parahippocampal gyrus, and increased connectivity in the left medial/superior temporal gyri and precentral gyrus within default mode network (DMN). De Bondt et al. (2015) also indicated that there were relationships between the premenstrual-like symptoms and the increased functional connectivity (FC) of the posterior part of the DMN with the precuneus, middle frontal gyrus, the posterior cingulate and cuneus. The above-mentioned studies show that neural abnormalities are embedded in PMS. However, the underlying elements leading to CNS dysfunctions in PMS are not well understood. Substantial studies have demonstrated that psychological changes may be the pivotal factors resulting in the brain activity abnormalities (Andermann, 1960; Walker and McGlone, 2013; Ait-Belgnaoui et al., 2014). Given that PMS patients are tested with significant psychological changes in menstrual cycle, especially at the luteal phase (Liu Q. et al., 2015; Watanabe and Shirakawa, 2015). The efforts to investigate the correlates between psychological changes and neural abnormalities may extend our understanding of the neural mechanism of PMS. While the amounts of literature concerning psychological processes on brain activity of PMS are limited and the studies to explicate the intricate neural mechanism of PMS are still insufficient.

Resting state functional magnetic resonance imaging (RS-fMRI) is a useful tool to gather further insight into intricate functions of human brain (Bifone and Gozzi, 2011; Branco et al., 2016). Regional homogeneity (ReHo), a data-driven method, measures the similarity or synchronization of the time series of nearest neighboring voxels, and can detect intensity of regional spontaneous brain activity at the resting state (Zang et al., 2004). Combining the RS-fMRI and ReHo method, researchers have detected abnormal neural activity in the resting state of neuropsychiatric disorders (Yuan et al., 2008), such as Parkinson’s disease (Wu et al., 2009), depression (Guo et al., 2011) and schizophrenia (Liu H. et al., 2006). These findings have confirmed the measurement reliability and sensitivity of ReHo in the neuropsychiatric fields, which may be helpful to investigate the abnormal brain activity in PMS.

Thereby, the aim of this study was to investigate the spontaneous brain activity in the women with PMS and healthy controls (HC) at the late luteal phase by ReHo method. We hypothesized that there existed significant changes of the spontaneous brain activity in women with PMS compared with HC. We also hypothesized that the neuroimaging findings could be associated with the psychological changes in PMS.

## Materials and Methods

### Ethics Statement

All subjects were informed about the whole experiment procedure and signed a written informed consent form. This study was approved by the Medicine Ethics Committee of First Affiliated Hospital, Guangxi University of Chinese Medicine, Guangxi, China. All the research procedures of the present study were conducted in accordance with the Declaration of Helsinki.

### Subjects

This study was performed on First Affiliated Hospital, Guangxi University of Chinese Medicine. Twenty-three patients (PMS-group) were recruited via advertisement in the Guangxi University of Chinese Medicine, Guangxi, China. To quantify premenstrual symptoms, all the patients were prospectively screened for 2 months and called for completing a daily record of severity of problems (DRSP) questionnaire decided by Dr. Endicott (Endicott et al., 2006; see Supplementary Material). Clinical diagnostic criteria for PMS were based on the recommendations and guidelines for PMS (Halbreich et al., 2007). Meanwhile, Diagnostic and Statistical Manual of Mental Disorders-5th Edition (DSM-5; American Psychiatric Association, 2013) was used to exclude patients from PMDD. All the patients were individually diagnosed by an experienced associated professor gynecologist. The inclusion criteria for PMS were met: (1) age ranged from 18 to 45 years old, being right-handed; (2) a regular menstrual cycle ranged from 24 to 35 days; (3) the premenstrual symptoms occurred up to 2 weeks before menses in most menstrual cycles; (4) symptoms remitted shortly following onset of menses and were absent during most of the mid-follicular phase of the menstrual cycle; (5) the symptoms were associated with impairment in daily functioning and/or relationships and/or caused suffering, such as emotional, behavioral and physical distress; (6) the menstrual-related cyclicity, occurrence during the late luteal phase of cycle (days −5 to −1) and absence during the middle follicular phase (days +6 to +10) were documented by repeated observations by the patients based on DRSP, and the mean luteal phase score was at least 30% greater than that of the follicular phase; and (7) the symptoms were not just an exacerbation or worsening of another mental or physical chronic disorders. The exclusion criteria for patients were as follows: (1) being currently pregnant or lactating; (2) having a history of thyroid disease, dysmenorrhea, gynecological inflammation, menopausal syndrome, hysterectomy or bilateral oophorectomy, mastopathy or cancer, or diabetes or any other structural diseases; (3) having psychiatric disorders by DSM-5 criteria, such as schizoaffective disorder, schizophrenia, organic mental disorder, delusional mental disorder, psychotic features coordinated or uncoordinated with mood or bipolar disorder; (4) treating with any steroid compound (including oral contraceptives and hormonal intrauterine devices), benzodiazepines, or other psychotropic drugs affecting PMS; (5) having any MRI contraindications; and (6) smoking or alcohol abuse.

Twenty-two age matched HC, right-handed women (HC group), with regular menstrual cycle of 24–35 days were recruited in this study. All the HC were free of psychiatric or neurological illness via assessment by medical history and physical examinations, and had no history of alcohol or drugs abuse. All the HC also underwent the same diagnostic screening tests.

Meanwhile, each subject was asked to complete an identical assessment protocol in the body mass index (BMI), women’s menstrual cycle, menophania, length of menstrual cycle, menstruation.

### Experimental Paradigm

The PMS group and HC group were randomly arranged to receive fMRI examinations. Based on the females’ physical characteristics and hormone level, all the test dates were set at the late luteal phase, ranging from 1 to 5 days before menstruation. To confirm the relatively stable and low level of endogenous cortisol and estradiol, all of the scan tests were conducted between 20:00 and 22:00 pm (Bao et al., 2004). For menstrual cycle stage verification, we obtained prospective self-reports about when their menstruation started and combined this information with the primary gynecological examinations and B-ultrasonic wave results to arrange the test times. The subject then received a RS-fMRI scan for 6 min. During the scan, each subject was instructed to keep eyes closed, not to think about anything and to stay awake.

### MRI Data Acquisition

MRI data were acquired using a 3.0 Tesla Siemens Magnetom Verio MRI System (Siemens Medical, Erlangen, Germany) at the Department of Radiology, First Affiliated Hospital, Guangxi University of Chinese Medicine, Nanning, Guangxi, China. To avoid head movement, each subject’s head was immobilized by foam pads in a standard 8-channel birdcage head coil. FMRI images were acquired with a single-shot gradient–recalled echo planar imaging (EPI) sequence with the parameters as following: repetition time (TR)/echo time (TE) = 2000 ms/30 ms, flip angle = 90°, field of view (FOV) = 240 mm × 240 mm, matrix size = 64 × 64, slice thickness = 5 mm and slices = 31. High resolution T1-weighted images were then obtained with a volumetric three-dimensional spoiled gradient recall sequence with the parameters as following: TR/TE = 1900 ms/2.22 ms, FOV = 250 mm × 250 mm, matrix size: 250 × 250, flip angle = 9°, slice thickness = 1 mm and 176 slices.

### Image Preprocessing

Preprocessing was performed with SPM8 (SPM8)^1^. The first 10 volumes of each functional time series were removed to avoid the instability of the initial MRI signal. The remaining images were corrected for acquisition time delay between different slices and realigned to the first volume. The head motion parameters were calculated by estimating the translation in every direction and the angular rotation on each axis for every volume. If the translation was more than 1.5 mm in any cardinal direction and the rotation was more than 1.5° in each of the orthogonal *x*, *y* and *z* axes, the subject was discarded. The realigned functional images were then spatially normalized to the Montreal neurological institute space using the normalization parameters estimated by T1 structural image unified segmentation, re-sampled to 3 mm × 3 mm × 3 mm voxels. Several sources of spurious variance, such as the estimated motion parameters, average blood oxygenation level dependent (BOLD) signals in ventricular and white matter regions, were dislodged from the images. After removing the variance, linear drift was removed and temporal filter (0.01–0.08 Hz) was then performed on the time series of each voxel to reduce the effect of low-frequency drifts and high-frequency noise.

### ReHo Analysis

The parameter of Kendall’s coefficient of concordance (KCC) was utilized to measure the similarity of time series of a given voxel to the ones of its 26 nearest voxels in a voxel-wise way based on the hypothesis that a voxel is temporally similar to the ones of its neighbors. Individual ReHo maps were created by computing KCC within a gray matter mask in a voxel-wise manner using REST Software^2^. When the center cube was on the edge of the gray matter mask, we only computed ReHo for a voxel if all of remaining nearest voxels were within the gray matter mask. For every subject, KCC map was normalized by dividing KCC in each voxel by the mean KCC of total gray matter. The KCC fMRI data were then spatially smoothed with a Gaussian kernel of 6 mm full-width at half-maximum.

### Statistical Analysis

Demographic and clinical data were compared using two-sample *t*-test. The threshold level in all statistical analysis for significance criterion was determined at *p* < 0.05. Two sample *t*-test was then applied to examine different patterns of the spontaneous brain activity between the PMS patients and HC. All of the contrast threshold was set at *p* < 0.05 (false discovery rate (FDR) corrected). We applied correlation analysis to estimate the relationships between the influence of symptom severity of disease and the regions of interest (ROIs) showing the differences in PMS patients compared with HC. First, the 6 mm sphere around the peak voxels in the significant between-group clusters was formed as the ROIs. Pearson correlations between the mean ReHo of the ROIs and DRSP values were then estimated. The age, BMI, menstruation, menophania, length of menstrual cycle were considered as covariates of no interest in this study. Adjustment for multiple comparisons was made with the Bonferroni correction for correlation analysis (*p* < 0.05).

## Results

### Demographic and Clinical Results

In this study, three PMS patients and one HC were excluded from further data analysis because of distinct head movement. Finally, 20 women with PMS and 21 matched HC were included in our study. There were no significant differences in terms of age, BMI, menstruation (days), menophania (years), length of menstrual cycle (days) between the PMS group and HC-group (Table 1).

**Table 1:** **Demographic and clinical characteristics for the study**.

Variable	PMS (*n* = 20)	HC (*n* = 21)	*p* value
Age (years)	21.85 ± 1.72	21.38 ± 0.86	0.284^a^
BMI	18.60 ± 1.71	19.50 ± 1.48	0.081^a^
Menophania (years)	13.75 ± 1.44	13.00 ± 1.09	0.068^a^
Length of menstrual cycle (days)	29.95 ± 1.76	29.80 ± 1.56	0.789^a^
Menstruation (days)	5.60 ± 1.09	5.38 ± 1.11	0.530^a^

*Abbreviations: PMS, premenstrual syndrome; HC, healthy control; BMI, body mass index. All values are mean ± standard deviation (SD). ^a^The p-value was obtained by two sample *t*-test*.

### DRSP Result

**Figure 1:**
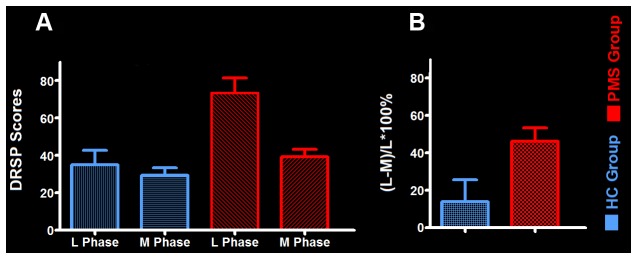
**The mean daily record of severity of problems (DRSP) score and its variation rate between groups. (A)** The mean DRSP score between PMS group and HC-group. **(B)** The variation rate of DRSP scores in PMS group and HC group. Abbreviations: PMS, premenstrual syndrome; HC, healthy control; L Phase, late luteal phase; M Phase, middle follicular phase.

The mean late luteal phase score of PMS group was 73.47 ± 7.84, it was the highest in groups. And the score variation rate from the middle follicular phase to the late luteal phase of PMS group was (46.24 ± 7.05)%. Each of the late luteal phase score in PMS group exceeded 50 and it was at least 30% larger than that of the follicular phase, which showed contrary to HC group (Table 2 and Figure 1).

**Table 2:** **The mean scores of late luteal phase and middle follicular phase and variation rate in PMS group and HC group**.

Groups	L Phase	M Phase	(L-M)/L*100%
**PMS group**
Subject 01	62.6	43.0	31.3%
Subject 02	67.0	35.2	47.5%
Subject 03	59.6	37.2	37.6%
Subject 04	66.2	39.6	40.2%
Subject 05	76.6	37.8	50.7%
Subject 06	88.0	46.6	47.0%
Subject 07	67.0	36.0	46.3%
Subject 08	86.8	32.0	63.1%
Subject 09	68.4	33.2	51.5%
Subject 10	65.6	40.6	38.1%
Subject 11	85.2	43.6	48.8%
Subject 12	74.6	40.4	45.8%
Subject 13	74.4	37.8	49.2%
Subject 14	78.8	45.0	42.9%
Subject 15	71.2	35.6	50.0%
Subject 16	73.0	38.2	47.7%
Subject 17	73.0	43.6	40.3%
Subject 18	80.8	35.2	56.4%
Subject 19	74.4	38.0	48.9%
Subject 20	76.2	44.6	41.5%
**HC group**
Subject 01	26.4	24.6	6.8%
Subject 02	43.0	30.4	29.3%
Subject 03	43.4	35.2	18.9%
Subject 04	31.0	32.0	−3.2%
Subject 05	43.8	29.6	32.4%
Subject 06	27.4	26.0	5.1%
Subject 07	34.4	30.6	11.0%
Subject 08	41.2	31.2	24.2%
Subject 09	28.4	27.6	2.8%
Subject 10	33.0	26.8	18.8%
Subject 11	42.4	30.0	29.2%
Subject 12	46.2	34.2	26.0%
Subject 13	26.8	25.8	3.7%
Subject 14	30.2	27.2	10.0%
Subject 15	24.8	24.0	3.2%
Subject 16	43.6	32.2	26.1%
Subject 17	24.0	25.2	−5.0%
Subject 18	28.2	25.4	9.9%
Subject 19	44.6	33.0	26.0%
Subject 20	40.6	39.4	3.0%
Subject 21	31.4	26.8	14.6%

*Abbreviations: PMS, premenstrual syndrome; HC, healthy control; L Phase, late luteal phase; M Phase, middle follicular phase*.

### Imaging Result

**Figure 2:**
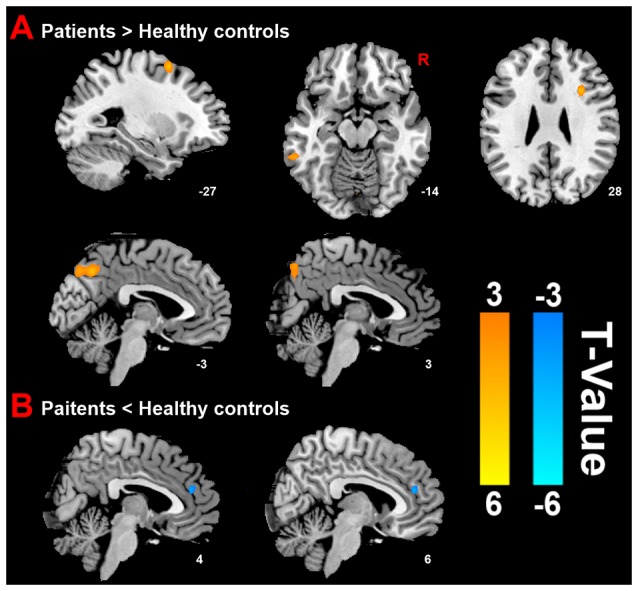
**Distinct brain regions. (A)** Increased regional homogeneity (ReHo) in brain regions between PMS patients and HC; **(B)** decreased ReHo in brain region between PMS patients and HC.

Compared with HC group, the results revealed that PMS group exhibited increased ReHo mainly in the bilateral precuneus, left inferior temporal cortex (ITC), right inferior frontal cortex (IFC) and left middle frontal cortex (MFC), as well as decreased ReHo in the right anterior cingulate cortex (ACC) at luteal phase (Figure 2).

### Correlation Analysis

**Figure 3:**
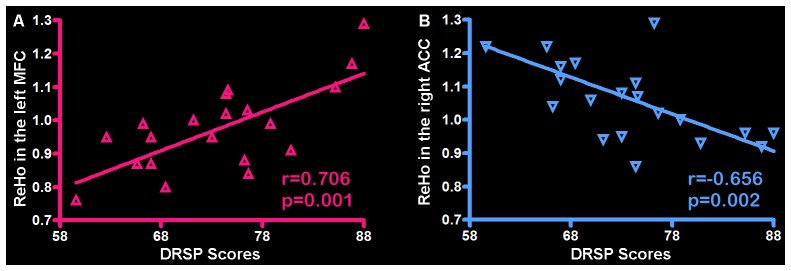
**Correlation between the ReHo and individual DRSP scores in the PMS group. (A)** The left MFC showed ReHo significant positive correlations with DRSP scores. **(B)** The right ACC showed ReHo significant negative correlations with DRSP scores. Each triangle represents the data from one subject. Abbreviation: DRSP, daily record of severity of problems; ReHo, Regional homogeneity; MFC, middle frontal cortex; ACC, anterior cingulate cortex.

Correlation analysis was applied to the ReHo and individual DRSP scores in the PMS group at luteal phase. The results indicated the DRSP scores in the PMS group positively correlated with the ReHo in the left MFC (*r* = 0.706, *p* = 0.001) and negatively correlated with the ReHo in the righ ACC (*r* = −0.656, *p* = 0.002; Figure 3).

## Discussion

In present study, we adopted a RS-MRI strategy to investigate the spontaneous neural activity in PMS patients based on the ReHo method. Compared with HC, we observed altered patterns of spontaneous neural activity in PMS at luteal phase, mainly located in the bilateral precuneus, left ITC, right IFC, left MFC and right ACC. Moreover, in PMS group, the left MFC and right ACC were associated with the severity of clinical symptoms based on DRSP.

The DRSP is considered to be a preferred instrument for prospective assessment on premenstrual disorders, it can provide the sensitive, reliable and valid measures of the symptoms and functional impairment criteria for PMS (Endicott et al., 2006). The evidence from previous study has demonstrated that the higher score of DRSP means PMS patients being at more negative and stressful conditions (Watanabe and Shirakawa, 2015). While psychological changes are deemed to make important impacts on the brain activity. Based on our findings of the highest DRSP scores at the late luteal phase in PMS group, we infer that patients might feel more stress and negative mood at the late luteal phase, such significant psychological changes might eventually affect the brain activity of PMS patients.

The first major finding of the present study was the decreased ReHo in right ACC of PMS patients compared with HC. ACC is well known to be one of the most important higher-order brain structure exceedingly related to emotional and cognitive processing (Mayberg et al., 2002; Davis et al., 2005; Lavin et al., 2013). ACC is also a central component in cognitive and execution control system referred to detect incongruence between expectations and outcomes during decision-making processes (Vincent et al., 2008). When human brains are suffered from the conflict monitoring in the relationships of the structure to other functional networks with which they interact may have important consequences for attention, affect and/or emotion regulation, ACC shows its central monitored and adjustive functions in effectively allocating the resources to the center (Kerns et al., 2004; Petersen et al., 2014). A meta-analysis pointed out that ACC was also a special structure vulnerably involved in generating emotional responses or expressing negative emotion, it showed functional dysregulation when frequently confronted negative emotional stimuli including fear, anxiety, pain and emotional conflict, and patients with generalized anxiety and stress disorders were related to the weakened or impaired functionality of ACC (Etkin et al., 2011). As far as we know, PMS patients were cyclically suffered from negative stimuli in emotion, physic and behavior. Thereby, our finding of decreased ReHo in ACC indicates that there might exist functional abnormality or impairment in ACC. The researchers reported that the negative symptoms had been shown to have important effects on the activity of ACC and influenced the behavior of functional networks at rest during the menstrual cycle (Petersen et al., 2014). A RS-fMRI study on women brains found that the gray matter volume in the ACC was significantly associated with menstrual cycle (De Bondt et al., 2013). The aforementioned literature results revealed that ACC was a vulnerable and crucial region referred to functional abnormality at the late luteal phase, which was correlated with the clinical negative symptoms. Based on the findings of DRSP scores negative correlation with ReHo and the right ACC, it suggests that PMS patients might be affected by negative psychological stimuli, and then the ACC lose its higher-order cognitive, controlled and executive functions or central monitored and adjustive functions in allocating the resources to the center, which may partly contribute to underlying neural mechanism of PMS. Nevertheless, PMS is a special syndrome cyclically recurring during the late luteal phase but relieving soon after the onset of menses (Tacani et al., 2015). We speculate that patients may be at the periodic states from in-control to out-control. Thereby, besides the ACC, there might be existed other important structures being responsible for the baseline brain activity of PMS patients.

The precuneus represents a relevant cortical structure of the parietal lobes. It is a crucial node of the DMN involved in imagery, simulation visuospatial integration and self-awareness. It is also a core region that is responsible for baseline brain activity, and participates in functions such as intrinsic ongoing mental processes and fundamental cognitive social functions (Pereira-Pedro and Bruner, 2016). The precuneus is associated with interoceptive and emotional processing with widely distributed networks sharing connectivity with many brain regions in the frontal, temporal, occipital and parietal cortices (Tanaka and Kirino, 2016). It showed greater activation to incongruent stimuli than to congruent stimuli (Kitada et al., 2014). Halbreich et al. (2003) pointed out that the CNS of PMS patients were usually suffered from incongruent stimuli rooted in the gonadal hormone’s fluctuations. Here, based on the finding of activation in the precuneus revealed by the increased ReHo, we speculate that this phenomenon may be due to the effect of incongruent stimuli on patients’ brain. Meanwhile, the negative symptoms may affect the precuneus and certain brain regions, and then the precuneus enhances its core action on maintaining the baseline brain activity or strengthens its networks connectivity with other brain regions. However, as to the healthy female subjects, studies showed the contrary results with decreased activation in precuneus during the luteal phase. Kunisato et al. (2011) reported that the precuneus would be inclined to work at morbid state during luteal phase (Helmbold et al., 2016). Thereby, due to the activation of precuneus, its higher-order functions may be motivated, and then the physical, emotional, cognitive and behavioral symptoms of PMS would be cyclically relieved soon after luteal phase. This phenomenon may be partly contributed to the functional adjustment of the precuneus in PMS patients. However, the development of PMS might be not merely confined to one region, the neural foundation of PMS would be an intricate process and might be involved in multiple regions, networks or system. There may have other dysfunctions of brain regions or functional networks in PMS patients.

Besides the finding of increased ReHo in the precuneus, our study showed increased ReHo in left ITC of PMS patients at luteal phase. Temporal cortex generally plays a crucial role in auditory, language processing and memory. However, previous studies have indicated that temporal cortex also serves as a key region in patients with psychiatric disorders because of abnormal activity or structure was found in the subregions of temporal cortex (Kasai et al., 2003; Ma et al., 2012). Halbreich pointed out that PMS might be related to abnormal temporal-limbic circuitry which might be heredity or acquired in a very early age (Halbreich, 2003). A SPECT study showed the changes of rCBF in the temporal lobes on luteal phase compared with the follicular phase in PMS patients (Buchpiguel et al., 2000). Liu Q. et al. (2015) demonstrated that PMS females showed increased FC in temporal cortex at luteal phase. Our observations were consistent with previous findings with abnormal activation in temporal cortex. In present study, PMS patients showed abnormal reactivity in the left ITC and right ACC (a region of limbic system) at the luteal phase compared with HC. It should be pointed out that further analysis using FC method is needed for the temporal-limbic emotional circuitry reactivity between the left ITC and right ACC, which might be more important to investigate the potential neural mechanism behind the emotion related disorder in female with PMS.

The abnormal changes in right IFC and left MFC were other interesting findings in PMS patients. IFC and MFC are two important parts of prefrontal cortex, which are taken part in integration of cognitive, emotional behaviors by uniting emotional biasing signals or markers into decision making processing (Gusnard et al., 2001; Simpson et al., 2001). The emotional response inhibition of prefrontal cortex was sensitive to the variations of the menstrual cycle (Amin et al., 2006), the observation from this study showed that there was significantly raised activation in the prefrontal cortex when faced to emotional response inhibition during the luteal phase by comparing with the follicular phase. This prior finding was consistent with our results of increased ReHo in right IFC and left MFC. Prefrontal cortex was a special brain functional region which could significantly express some characteristic receptors contributed to the symptoms of PMS. Previous literature showed that prefrontal cortex enriched the CNS-related receptors concentration (Kugaya et al., 2003; Liu B. et al., 2015). These receptors have intrinsic influence on cognitive and emotional functions in the menstrual cycle (Bethea et al., 2000). It will further provide the neural base for explicating the right IFC and left MFC involved in PMS. Not coincidentally, as the sever form of PMS, PMDD patients were found similar abnormalities in prefrontal cortex. For example, Gingnell et al. (2013) showed that there was significantly increased reactivity in the prefrontal cortex when referred to negative emotional stimuli during the luteal phase. Moreover, PMDD patients had greater prefrontal activation than healthy subjects, and the abnormally increased activation of prefrontal cortex was correlated with the degree of the disease (Baller et al., 2013). Based on the finding of the DRSP scores positive correlation with ReHo in the left MFC, it could indicate that the left MFC might be another key region related to the degree symptom of PMS. Nevertheless, our observations were inconsistent with the findings of Liu Q. et al. (2015), the study demonstrated that the PMS patients had decreased activity in the MFC. We inferred that these incomplete coincidences with each other might be attributed to the different sample or analysis method, yet the MFC as an important region involving in the abnormal brain activity for the PMS patients was consensual. The increased ReHo in right IFC and left MFC suggests that the cortical emotional circuitry reactivity during negative stimuli is altered in PMS at the luteal phase, which might be part of the pathophysiology behind the emotional and cognitive symptoms or lack of emotional control reported by female with PMS. And the abnormalities within the right IFC and left MFC are speculated to hinder the processing of brain function and therefore to constitute a vicious cycle in the maintenance of clinical manifestations of PMS.

However, in holistic perspective, abnormal functional areas in PMS may go beyond the functions of single areas due to these areas usually interacting with each other to provide a more comprehensive brain system. In present study, we found the changed brain areas including the precuneus, ITC, ACC, IFC and MFC, which were referred to the default system functions. The DMN plays a vital role in self-referential activity, such as assessing characteristics of external and internal cues, planning the future, and remembering the past (Raichle and Snyder, 2007; Buckner et al., 2008). More importantly, theoretical models concerning the role of the default system in psychopathology had been well described in the previous literature (Zhao et al., 2007; Sheline et al., 2009; Messina et al., 2016). Given that psychological changes indeed occur in PMS, we conclude that the psychological changes might have some effects on modulating activity of DMN in PMS patients during the luteal phase. Our conclusion is in accordance with previous studies (De Bondt et al., 2015; Liu Q. et al., 2015), which observed the abnormal activity in the DMN for PMS patients. Thereby, the changed DMN would attach much more importance to the neural mechanism of PMS.

Our study was subject to some limitations that need to be taken into consideration: (1) the luteal phase was a key stage involved in PMS, PMS patients mainly referred to a series of symptoms in this phase. So, our study merely made an investigation on the abnormal brain activity of PMS patients at luteal phase by comparing with HC. The study did not investigate whether or not abnormal brain activity might exist among the luteal phase and follicular phase in PMS group and HC group, we would pay more concern on these topics in next stage. (2) Another limitation of the present study was the relatively limited sample size. Enlargement of the sample size would be the direction of our future study; and (3) The method of ReHo can not directly show the connectivity between brain regions, further analysis and application by diverse data analysis methods (e.g., FC) or multi modal functional magnetic resonance imaging may extend our understanding of the neural mechanism of PMS.

## Conclusion

To summarize, we observed the changed patterns of spontaneous neural activity in PMS patients during the resting state, with increased ReHo in the bilateral precuneus, left ITC, right IFC, left MFC and decreased ReHo in the right ACC at luteal phase. Furthermore, our findings showed that the DRSP scores (an important index for assessing PMS severity and symptoms) in the PMS group positively correlated with ReHo in the left MFC and negatively correlated with the ReHo in the right ACC. The present findings may enhance our understanding of the neurobiological underpinnings of PMS.

## Author Contributions

DD made substantial contributions to the overall conception of the work, designed the experiment, revised and handled the manuscript, accurately answered all the questions from the reviewers, ensured the integrity of the work and approved the final version to be published. HLiao made important contribution to the literature review, interpreted data for the work, drafted the manuscript, revised some critical structure and intellectual content, advised on the integrity of the work and approved the final version to be published. YP provided the whole designed theory of the study, made substantial contributions to the overall design and guideline of experiment, interpreted intellectual content, substantial work on drafting the manuscript and approved the final version to be published. PL conducted the data processing, interpreted intellectual content in disease, was accountable for some aspects of the work in ensuring that questions related to the accuracy of the work were appropriately resolved and approved the final version to be published. HLiu executed the diagnosis and assessment of patients, recruited subjects for this study, advised on the improvement of protocols, provided important intellectual content in experiment and approved the final version to be published. GD made important contribution to the design of MR scan protocols, carried out MRI operation, MRI data acquisition and storage and approved the final version to be published. YL conducted the data processing and analysis for the functional MRI data, interpreted the conceptions of data processing, wrote the procedure of data processing and approved the final version to be published. LT, JT, DW, SL and LL recruited subjects for this study, conducted the assessment the symptom, diagnosis and treatment of the patients, gave some important advices for the accuracy interpretation or description of conceptions related to the disease and approved the final version to be published.

## Conflict of Interest Statement

The authors declare that the research was conducted in the absence of any commercial or financial relationships that could be construed as a potential conflict of interest.

## Footnotes

^1^http://www.fil.ion.ucl.ac.uk/spm/

^2^http://restfmri.net/forum/index.php

## Abbreviations

ACC: Anterior cingulate cortex
BMI: Body mass index
BOLD: Blood oxygenation level dependent
CNS: Central nervous system
DMN: Default mode network
DSM-5: Diagnostic and Statistical Manual of Mental Disorders-5th Edition
DRSP: Daily rating of severity of problems
EPI: Echo planar imaging
FC: Functional connectivity
FDR: False discovery rate
FOV: Field of view
HC: Healthy controls
IFC: Inferior frontal cortex
ITC: Inferior temporal cortex
KCC: Kendall’s coefficient of concordance
MFC: Middle frontal cortex
PMDD: Premenstrual dysphoric disorder
PMS: Premenstrual syndrome
rCBF: regional cerebral blood flow
ReHo: Regional homogeneity
ROIs: Regions of interest
RS-fMRI: Resting state functional magnetic resonance imaging
SPECT: Single photon emission computed tomography
TE: Echo time
TR: Repetition time.
