# Supplementary material for: Abnormal Spontaneous Brain Activity in Women with Premenstrual Syndrome Revealed by Regional Homogeneity
Source: Front Hum Neurosci. 2017 Feb 13;11:62. doi: 10.3389/fnhum.2017.00062 (PMC5303726; doi:10.3389/fnhum.2017.00062)
Supplement: Supplementary file 1 [file Presentation_1.pdf]

Supplementary Material

Abnormal spontaneous brain activity in Women with Premenstrual Syndrome revealed by regional homogeneity

Hai Liao[1†], Yong Pang[2†], Peng Liu[3], Huimei Liu[2], Gaoxiong Duan[1], Yanfei Liu[3], LijunTang[2], Jien Tao[2], Danhong Wen[4], Shasha Li[1], Lingyan Liang[1], and Demao Deng[1]*

[1]Department of Radiology, First Affiliated Hospital, Guangxi University of Chinese Medicine, Nanning 530023, Guangxi, China
[2]Department of Acupuncture, First Affiliated Hospital, Guangxi University of Chinese Medicine, Nanning 530023, Guangxi, China
[3]Life Science Research Center, School of Life Science and Technology, Xidian University, Xi'an 710071, Shaanxi , China
[4]Department of Teaching, First Affiliated Hospital, Guangxi University of Chinese Medicine, Nanning, Guangxi 530023, China

[†] These authors contributed equally to this work.

*** Correspondence:**
Full Name: Demao Deng
Postal Address: Department of Radiology, First Affiliated Hospital, Guangxi University of Chinese Medicine, Nanning, Guangxi 530023, China
E-mail: demaodeng@163.com
Tel: +86 0771 5848702

All prospective participants were given the DRSP for completing every night over the two months before the initial fMRI scanning. Women were asked to give a score of 1 to 6 for each symptom according to its severity. The levels of severity on the DRSP are: 1-Not at all, 2-Minimal, 3-Mild, 4-Moderate, 5-Severe, 6-Extreme.

1a. Felt depressed, sad, "down," or "blue"
1b. Felt hopeless
1c. Felt worthless, or guilty
2. Felt anxious, tense, "keyed up" or "on edge"
3a. Had mood swings (e.g., suddenly felt sad or tearful)
3b. Was more sensitive to rejection or my feelings were easily hurt
4a. Felt angry, irritable
4b. Had conflicts or problems with people
5. Had less interest in usual activities (e.g., work, school, friends, hobbies)
6. Had difficulty concentrating
7. Felt lethargic, tired, fatigued, or had a lack of energy
8a. Had increased appetite or overate
8b. Had cravings for specific foods
9a. Slept more, took naps, found it hard to get up when intended
9b. Had trouble getting to sleep or staying asleep
10a. Felt overwhelmed or that I could not cope
10b. Felt out of control
11a. Had breast tenderness
11b. Had breast swelling, felt "bloated", or had weight gain
11c. Had headache
11d. Had joint or muscle pain

At work, at school, at home, or in daily routine, at least one of the problems noted above caused reduction of productivity or inefficiency

At least one of the problems noted above interfered with hobbies or social activities (e.g., avoid or do less)

At least one of the problems noted above interfered with relationships with others.

The diagnosis of PMS was established when the mean score 5 days before menstruation exceeded 50, the mean luteal phase score was at least 30% greater than that of the follicular phase, and at least 3 symptoms scored more than 3 in the 2 months.
